# Galanin in an Agnathan: Precursor Identification and Localisation of Expression in the Brain of the Sea Lamprey *Petromyzon marinus*

**DOI:** 10.3389/fnana.2019.00083

**Published:** 2019-09-13

**Authors:** Daniel Sobrido-Cameán, Luis Alfonso Yáñez-Guerra, Francesco Lamanna, Candela Conde-Fernández, Henrik Kaessmann, Maurice R. Elphick, Ramón Anadón, María Celina Rodicio, Antón Barreiro-Iglesias

**Affiliations:** ^1^Department of Functional Biology, CIBUS, Faculty of Biology, Universidade de Santiago de Compostela, Santiago de Compostela, Spain; ^2^School of Biological and Chemical Sciences, Queen Mary University of London, London, United Kingdom; ^3^Center for Molecular Biology of Heidelberg University (ZMBH), DKFZ-ZMBH Alliance, Heidelberg, Germany

**Keywords:** lamprey, galanin, telencephalon, hypothalamus, striatum, neuropeptides

## Abstract

Galanin is a neuropeptide that is widely expressed in the mammalian brain, where it regulates many physiological processes, including feeding and nociception. Galanin has been characterized extensively in jawed vertebrates (gnathostomes), but little is known about the galanin system in the most ancient extant vertebrate class, the jawless vertebrates or agnathans. Here, we identified and cloned a cDNA encoding the sea lamprey (*Petromyzon marinus*) galanin precursor (*PmGalP*). Sequence analysis revealed that *PmGalP* gives rise to two neuropeptides that are similar to gnathostome galanins and galanin message-associated peptides. Using mRNA *in situ* hybridization, the distribution of *PmGalP*-expressing neurons was mapped in the brain of larval and adult sea lampreys. This revealed *PmGalP-*expressing neurons in the septum, preoptic region, striatum, hypothalamus, prethalamus, and displaced cells in lateral areas of the telencephalon and diencephalon. In adults, the laterally migrated *PmGalP*-expressing neurons are observed in an area that extends from the ventral pallium to the lateral hypothalamus and prethalamus. The striatal and laterally migrated *PmGalP-*expressing cells of the telencephalon were not observed in larvae. Comparison with studies on jawed vertebrates reveals that the presence of septal and hypothalamic galanin-expressing neuronal populations is highly conserved in vertebrates. However, compared to mammals, there is a more restricted pattern of expression of the galanin transcript in the brain of lampreys. This work provides important new information on the early evolution of the galanin system in vertebrates and provides a genetic and neuroanatomical basis for functional analyses of the galanin system in lampreys.

## Introduction

The neuropetide galanin was named as such because in most species it contains an N-terminal glycine and a C-terminal alanine (Tatemoto et al., 1983). The mature galanin peptide comprises 29–30 residues and is cleaved from a pro-peptide precursor that also generates the longer galanin message-associated peptide (GMAP; 60 residues in humans). The N-terminal part of the mature galanin peptide is crucial for its biological activity and is highly conserved in jawed vertebrates. Galanin is expressed in the central and peripheral nervous systems and signals via three receptor subtypes to regulate many physiological processes, including feeding, arousal/sleep, learning and memory, pituitary hormone release, nerve regeneration, stress/anxiety, nociception/pain and thermoregulation (for reviews see Lang et al., 2007, 2015; Šípková et al., 2017).

The galanin pro-peptide has been identified biochemically or genetically in many jawed vertebrates, including mammalian and non-mammalian species, and the galaninergic system has been extensively characterized in the brain of jawed vertebrates (for reviews see Mensah et al., 2010; Lang et al., 2015). In mammals, including humans, galanin is widely expressed in the brain with galanin-expressing neuronal populations present in the telencephalon, hypothalamus and brainstem (Rökaeus et al., 1984; Skofitsch and Jacobowitz, 1985; Kaplan et al., 1988; Cortés et al., 1990; Elmquist et al., 1992; Kordower et al., 1992; Palkovits and Horváth, 1994; Cheung et al., 2001; Pérez et al., 2001; for a review see Jacobowitz et al., 2004). In amphibians, reptiles and birds, the telencephalon, hypothalamus, mesencephalon and rhombencephalon also contain galanin-expressing neurons (Lázár et al., 1991; Olivereau and Olivereau, 1992; Józsa and Mess, 1993; Jiménez et al., 1994). However, in fishes the expression of galanin appears to be more restricted to telencephalic and hypothalamic areas (Vallarino et al., 1991; Unniappan et al., 2004; Adrio et al., 2005; Rodríguez Díaz et al., 2011; for a review see Mensah et al., 2010).

In contrast to jawed vertebrates, there is very little information on the galanin system of jawless vertebrates or agnathans, which include lampreys. Agnathans occupy a key phylogenetic position at the base of the vertebrate tree, which makes them interesting models to understand the early evolution of neuropeptidergic systems in vertebrates. In addition, lampreys have complex life cycles with very different larval and adult stages in terms of their anatomy and feeding behavior, which provides an excellent model to understand the roles that a given neuropeptidergic system plays in different behavioral circumstances in the same species.

Only a few studies have looked at the organization of the galanin system in lampreys (Buchanan et al., 1987; Jiménez et al., 1996; Pombal and Puelles, 1999; Yáñez et al., 1999; Bosi et al., 2004). These studies were conducted using antibodies generated against porcine galanin and reported the distribution of galanin-like immunoreactivity in the spinal cord (Buchanan et al., 1987), brain (Jiménez et al., 1996), and parapineal organ (Yáñez et al., 1999) of adult lampreys. Galanin-like-immunoreactive (ir) fibers, but not immunoreactive neurons, are present in the spinal cord of adult lampreys, mainly in its lateral region (Buchanan et al., 1987). In the brain, galanin-like-ir neurons are present in the telencephalon, hypothalamus and prethalamus, but not in the mesencephalon or rhombencephalon (Jiménez et al., 1996). Galanin-like-ir fibers have been also described in different brain regions, including the prosencephalon, mesencephalon and rhombencephalon (Jiménez et al., 1996), and the parapineal organ (Yáñez et al., 1999) of adult lampreys. However, the galanin precursor transcript/peptide has not yet been identified in lampreys and the roles of galanin in the sea lamprey CNS are not known.

Here, we report the identification of the galanin precursor transcript of the sea lamprey *Petromyzon marinus* (*PmGalP*). Sequence analyses revealed that this pro-peptide contains galanin and GMAP peptide sequences. We also report the pattern of expression of *PmGalP* in the CNS of both larval and adult animals by means of *in situ* hybridization (ISH). Our results confirmed the presence of the known galanin-expressing periventricular neuronal populations of lampreys, but we also discovered the existence of other *PmGalP-*expressing neuronal populations, including the presence of laterally migrated neurons in the diencephalon and hypothalamus. Our results provide a genetic and neuroanatomical basis for future functional studies on the role of galanin and GMAP in the CNS of lampreys.

## Materials and Methods

### Animals

Larval (*n* = 10) and adult (downstream migrating young adults, *n* = 2; upstream migrating adults, *n* = 3) sea lampreys, *P. marinus* L., were used for this study. Downstream migrating young adults and larvae (ammocoete: lengths comprised between 80 and 120 mm, 4–7 years old) were collected from the River Ulla (Galicia, Spain) with permission from the *Xunta de Galicia*. Upstream migrating adults were acquired from local suppliers. Adults were fixed freshly, and larvae were maintained in aquaria containing river sediment and with appropriate feeding, aeration and temperature conditions until the day of use. Before all experiments, animals were deeply anesthetized with 0.1% tricaine methanesulfonate (MS-222; Sigma, St. Louis, MO, United States) in fresh water and killed by decapitation. All experiments were approved by the Bioethics Committee at the University of Santiago de Compostela and the *Conselleriìa do Medio Rural e do Mar* of the *Xunta de Galicia* (License Ref. JLPV/IId) and were performed in accordance with European Union and Spanish guidelines on animal care and experimentation.

### Cloning and Sequencing of the *PmGalP* cDNA

The *PmGalP* sequence was identified in a custom annotation of protein-coding genes (unpublished data) based on the *P. marinus* germline genome (Smith et al., 2018). This sequence was deposited in GenBank under accession number MK977616.

Larvae (*n* = 5) were anesthetized as indicated above and the brain and spinal cord were dissected out under sterile conditions. Total RNA was isolated from these tissues using the TriPure reagent (Roche, Mannheim, Germany). The first-strand cDNA synthesis reaction from total RNA was catalyzed with Superscript III reverse transcriptase (Invitrogen, Waltham, MA, United States) using random primers (hexamers; Invitrogen). For polymerase chain reaction (PCR) cloning, specific oligonucleotide primers (forward: 5′-TCTGCGTGCCATCATCGACT-3′; reverse: 5′-TTACGCTTAGCTCGCCACGA-3′) were designed based on the *PmGalP* transcript sequence. The amplified fragments were cloned into pGEM-T easy vectors (Promega, Madison, WI, United States) using standard protocols and sequenced by GATC Biotech (Cologne, Germany) using Sanger sequencing, which confirmed the original sequence.

### Alignment of the *PmGalP* Sequence With Galanin Precursor Sequences From Other Vertebrates and Phylogenetic Analyses

**FIGURE 1:**
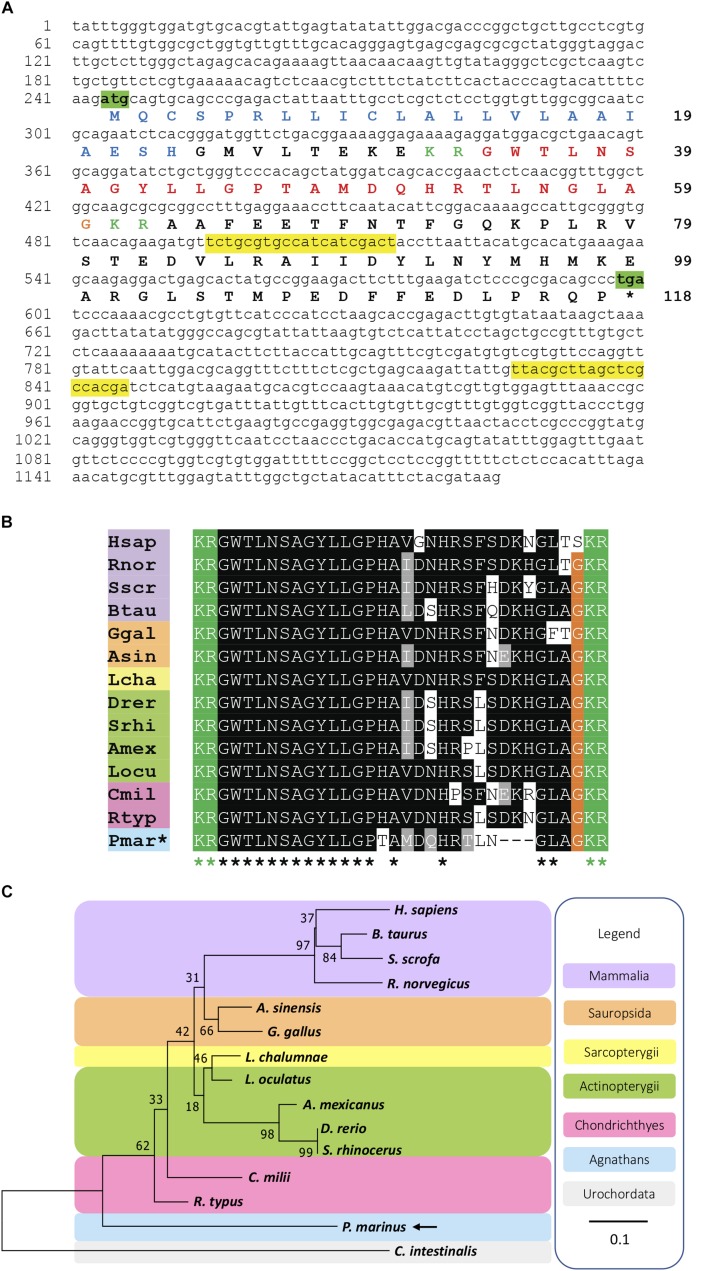
Identification of a galanin precursor in the sea lamprey *Petromyzon marinus.* **(A)** Nucleotide sequence (lower case) of a transcript that encodes the *Petromyzon marinus* galanin precursor (PmGalP; upper case). The start and stop codons are highlighted in green. The predicted signal peptide sequence is shown in blue and dibasic cleavage sites are shown in green. The putative galanin peptide derived from the precursor protein is shown in red, with the C-terminal glycine that is substrate for amidation shown in orange. The primers used for cloning of a fragment of *PmGalP* cDNA are highlighted in yellow. **(B)** Alignment of a region of PmGalP, including the galanin peptide bounded by dibasic cleavage sites, with the corresponding region of galanin precursor proteins from other vertebrate species. Conserved residues are highlighted, with conservation in more than 70% of sequences shown in black and with conservative substitutions shown in gray. **(C)** Neighbor-joining tree showing relationships of galanin-type precursors in selected chordate species. The percentage of replicate trees in which the associated taxa clustered together in the bootstrap test (1000 replicates) are shown next to the branches. The analysis was conducted in MEGA 7. The urochordate galanin-like sequence from *Ciona intestinalis* (Cint) was used to root the tree and is highlighted in gray. Species names in the alignment **(B)** are as follows: Hsap (*Homo sapiens*), Btau (*Bos taurus*), Rnor (*Rattus norvegicus*), Sscr (*Sus scrofa*), Ggal (*Gallus gallus*), Asin (*Alligator sinensis*), Lcha (*Latimeria chalumnae*), Srhi (*Sinocyclocheilus rhinocerous*), Drer (*Danio rerio*), Amex *(Astyanax mexicanus)*, Locu *(Lepisosteus oculatus)*, Rtyp *(Rhincodon typus)*, Cmil (*Callorhinchus milii*), Pmar (*Petromyzon marinus*). Additionally, in the alignment **(B)** and the phylogenetic tree **(C)**, species names are highlighted in taxon-specific colors: purple (mammals), orange (sauropsids), yellow (lobe-finned fishes), green (ray-finned fishes), pink (cartilaginous fishes), blue (agnathans). The accession numbers and the alignment of the sequences used to build this phylogenetic tree are shown in Supplementary File S1.

The amino acid sequence of the PmGalP (GenBank; MK977616) was obtained by translation of the cDNA sequence using ExPASy (Gasteiger et al., 2003), and the signal peptide was predicted using SignalP 4.0 (Petersen et al., 2011). The PmGalP sequence was aligned with galanin precursors from a variety of vertebrate species, including mammals, sauropsids, lobe-finned fishes, ray-finned fishes, and cartilaginous fishes (see section “Supplementary File S1” for a list of the sequences used). The alignments shown in Figure 1B and Supplementary Figure S1 were performed using MAFFT (Katoh et al., 2017), with the number of maximum iterations set to 1000 to ensure an optimal alignment. The scoring matrix used was BLOSUM62. The alignment generated was highlighted using the software BOXSHADE^1^ with 80% conservation as the minimum. Finally, the sequences were highlighted in phylum-specific colors: mammals (purple), sauropsids (orange), lobe-finned fishes (yellow), ray-finned fishes (green), cartilaginous fishes (pink), and agnathans (blue).

A phylogenetic analysis of galanin precursors was performed using the Neighbor-Joining method (Saitou and Nei, 1987). The amino acid sequences of full- length precursors (see section “Supplementary File S1” for a list of the sequences) were aligned using MAFFTT and a tree was generated, the *Ciona intestinalis* galanin-like peptide precursor was designated as an outgroup. The percentage of replicate trees in which the associated taxa clustered together in the bootstrap (Efron et al., 1996) test (1000 replicates) are shown next to the branches. The substitution model used was Jones-Taylor-Thornton Gamma distributed. The tree is drawn to scale, with branch lengths in the same units as those of the evolutionary distances used to infer the phylogenetic tree. The phylogenetic analysis was conducted in MEGA7 (Kumar et al., 2016).

### *In situ* Hybridisation

Templates for *in vitro* transcription were prepared by PCR amplification as follows. A 352-base pair (bp) fragment of the *PmGalP* sequence was obtained using the primers described but in this case, the reverse primer included the sequence of the universal T7 promoter (TAAGCTTTAATACGACTCACTATAGGGAGA). For the generation of sense probes, the sequence of the T7 promoter was included in the forward primers. Digoxigenin (DIG)-labeled riboprobes were synthesized using the amplified fragments as templates and following standard protocols using a T7 polymerase (Nzytech, Lisbon, Portugal).

The methods employed for mRNA *in situ* hybridisation were the same as previously described for tyrosine hydroxylase, a 5-HT1a receptor and a GABA_*B*_ receptor (Barreiro-Iglesias et al., 2010; Cornide-Petronio et al., 2013; Romaus-Sanjurjo et al., 2016). Briefly, the brains/rostral spinal cords of larvae and young and mature adults were dissected out and fixed by immersion for 12 h in 4% paraformaldehyde (PFA) in phosphate-buffered saline (PBS) at 4°C. Then, they were cryoprotected with 30% sucrose in PBS, embedded in Tissue-Tek^®^ O.C.T.^TM^ Compound (Sakura, Torrance, CA, United States), frozen in liquid nitrogen-cooled isopentane, and cut serially on a cryostat (14μm thickness) in transverse planes. Sections were mounted on Superfrost^®^ Plus glass slides (Menzel, Brunswick, Germany). The sections were incubated with the *PmGalP* DIG-labeled antisense riboprobe (1μg/mL) at 70°C overnight in hybridization mix and treated with RNAse A (Sigma) in the post-hybridization washes. Then, the sections were incubated with a sheep anti-DIG antibody conjugated to alkaline phosphatase (1:2000; Roche) overnight at 4°C. Staining was conducted in BM Purple (Roche) at 37°C until the signal was clearly visible. No staining was detected when using sense probes. Finally, the sections were mounted in Mowiol^®^ (Sigma).

### Imaging

Photomicrographs were obtained with an BX51 microscope equipped with a DP71 digital camera (Olympus, Tokyo, Japan). Plates of photomicrographs and minimal bright/contrast adjustments were performed with Photoshop CS (Adobe). Drawings were done with CorelDraw 2019.

### Nomenclature

For the nomenclature of brain regions and brain nuclei we followed the nomenclature used by our group in recent studies on the organization of different neuronal systems (including neuropeptidergic systems) in the sea lamprey brain (Barreiro-Iglesias et al., 2017; Fernández-López et al., 2017). In some instances, equivalencies to nomenclatures used by other authors are mentioned in the results and discussion. The readers should take into account that in lampreys most mature neurons are located in periventricular locations in the brain and do not migrate away from the ventricle.

## Results

### Identification of *PmGalP* and Sequence Analysis

Analysis of *P. marinus* germline genome sequence data revealed the occurrence of a candidate galanin precursor in *P. marinus* (*PmGalP*; GenBank accession number MK977616). PmGalP is a 118-residue protein (Figure 1) with a 23-residue signal peptide, a 26 residue galanin-like peptide bounded by dibasic cleavage sites (Figure 1A) and a 56-residue galanin-associated peptide-like sequence that spans from the second dibasic cleavage site to the C-terminus of the precursor (Supplementary Figure S1).

The sequence of the predicted C-terminally amidated mature peptide was aligned with galanin-type peptides from other vertebrates, including mammals, sauropsids, lobe-finned fishes, ray-finned fishes, and cartilaginous fishes. Comparison of the *P. marinus* galanin with gnathostome galanins revealed both similarities and differences. Comprising 26 residues, *P. marinus* galanin is shorter than gnathostome galanins, which are 29 or 30 residues in length (Figures 1A,B). However, the first thirteen residues of *P. marinus* galanin are identical to gnathostome galanins (Figure 1B). The residue at position 14 (histidine, H) is conserved in all gnathostome galanins, whereas in *P. marinus* galanin this position is occupied by a threonine (T) residue, which is a non-conservative substitution. Positions 15 to 21 in *P. marinus* galanin have conservative substitutions with respect to gnathostome galanins, but by comparison with human galanin positions 22 and 23 in *P. marinus* galanin have non-conservative substitutions of Phenylalanine (F) with Leucine (L), and of Serine (S) with Asparagine (N), respectively. However, this feature is not unique to *P. marinus* galanin, because differences at position 22 are also seen in all the ray-finned fishes and in the cartilaginous fish *Rhincodon typus* and differences at position 23 are also seen in two sauropsids and in the cartilaginous fish *Callorhinchus milii*. Residues at positions 24 to 26 in gnathostome galanins are missing in *P. marinus* galanin but the C-terminal GLAamide of *P. marinus* galanin is a highly conserved feature of most gnathostome galanins (Figure 1B).

Based on an alignment of PmGalP with fourteen other galanin-type precursor protein sequences, a phylogenetic reconstruction was made using the neighbor-joining method with the galanin-type precursor from the urochordate *C. intestinalis* used to root the tree. The phylogenetic analysis of precursors shows that the PmGalP occupies a position in the tree consistent with the basal phylogenetic position of agnathans in vertebrate phylogeny (Figure 1C).

### Distribution of *PmGalP*-Expressing Neuronal Populations in the Lamprey Brain

**FIGURE 2:**
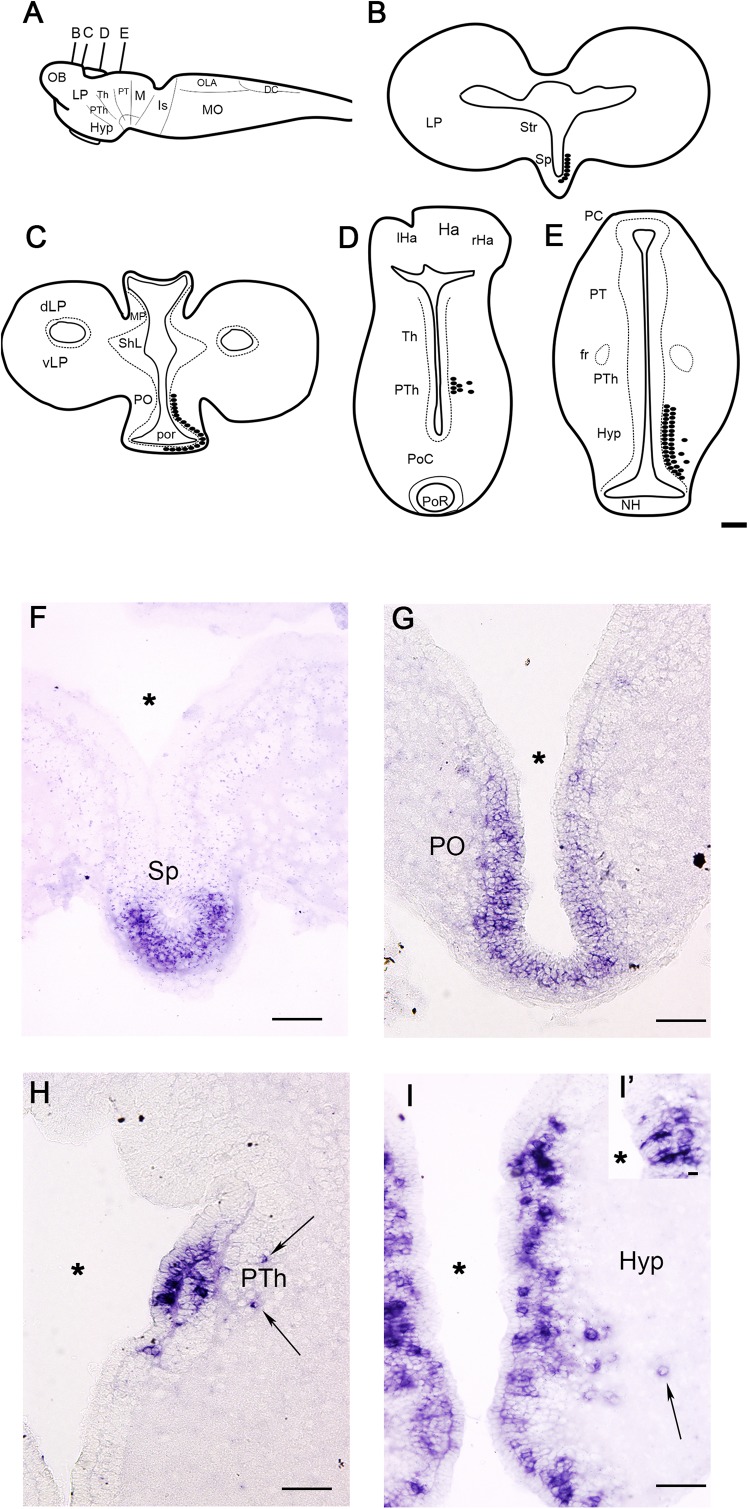
Schematic drawings **(A–E)** and photomicrographs **(F–I)** of sections of the larval sea lamprey brain showing the distribution of *PmGalP* expressing neurons. For abbreviations, see list. The plane of section of schematic drawings **B–E** is indicated in **A**. Arrows indicate the presence of laterally migrated cells. The asterisks indicate the ventricles. A detail of CSF-c cells of the hypothalamus is shown in **I’**. Dorsal is to the top. Scale bars: 100 μm.

**FIGURE 3:**
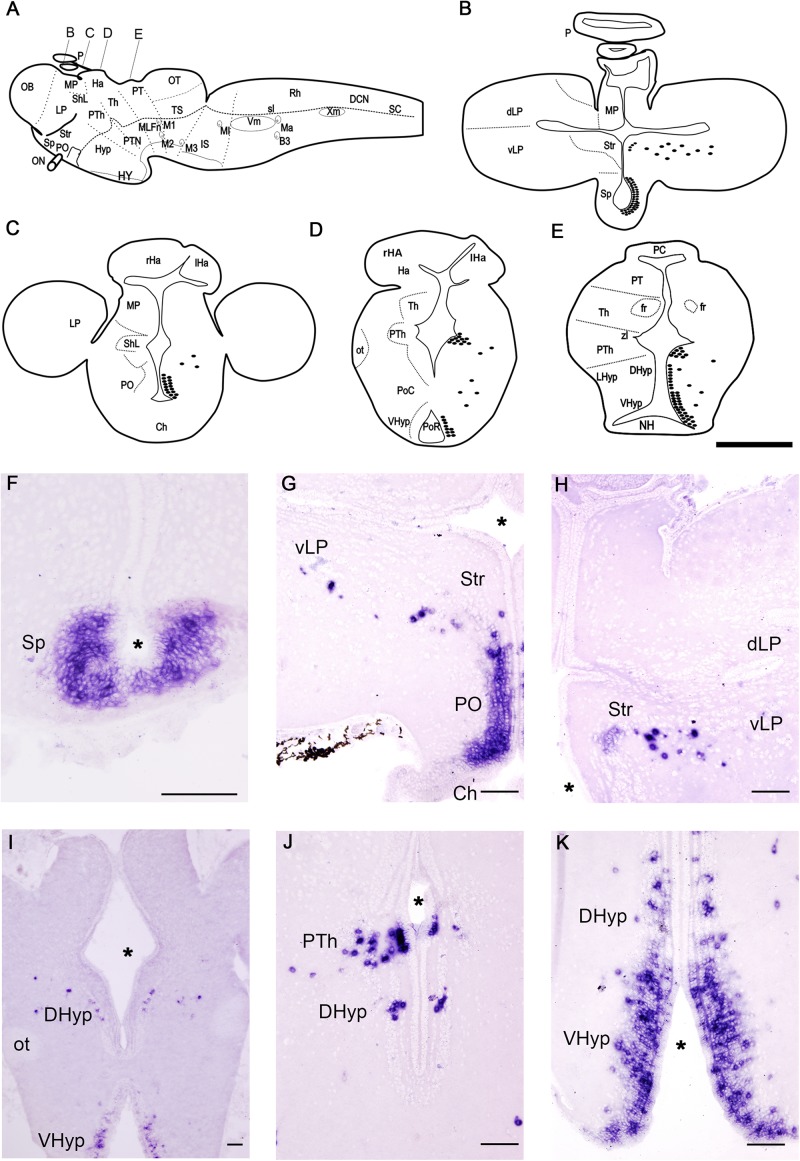
Schematic drawings **(A–E)** and photomicrographs **(F–K)** of sections of the adult sea lamprey brain showing the distribution of *PmGalP* expressing neurons. For abbreviations, see list. **I** is a photomicrograph of an upstream migrating adult sea lamprey, the rest of the photomicrographs are from young adults. The plane of section of schematic drawings **B–E** is indicated in **A**. The asterisks indicate the ventricles. Dorsal is to the top. Scale bars: 100 μm.

The expression of the *PmGalP* transcript in the CNS of the sea lamprey was analyzed using mRNA *in situ* hybridisation. Expression of *PmGalP* was restricted to the prosencephalon and no expression was detected in the mesencephalon, rhombencephalon or spinal cord of both larval (Figure 2) and adult (Figure 3) sea lampreys.

### Larvae

The distribution of *PmGalP*-positive (*PmGalP+*) neurons was analyzed in larvae with body lengths between 80 and 120 mm (Figure 2). *PmGalP+* neurons were found in two telencephalic regions (Figures 2B,C,F,G). The most rostral population of *PmGalP+* cells was found in a periventricular location in the septum (septocommissural preoptic area of Pombal et al., 2009; Figures 2B,F). Strongly stained *PmGalP+* neurons were also found in the preoptic nucleus (Figures 2C,G). This preoptic population appeared as a caudal continuation of the septal population.

In the alar diencephalon, a group of strongly stained *PmGalP+* cells was observed in the rostral part of the prethalamus (prosomere 3; see Pombal et al., 2009). In this region, most of the *PmGalP+* cells are located in the periventricular area (Figures 2D,H), but some laterally displaced *PmGalP+* cells were also observed (Figures 2D,H). In the hypothalamus, numerous *PmGalP+* cells were observed in the periventricular area of the infundibular recess (ventral hypothalamus; Figures 2E,I). Some of these cells showed a strongly stained dendrite crossing the ependymal layer, suggesting that they are cerebrospinal fluid-contacting cells (Figure 2I). Some laterally displaced *PmGalP+* cells were also present in this hypothalamic region (Figures 2E,I).

### Adults

We investigated possible changes in the *PmGalP+* populations after metamorphosis and during sexual maturation by analyzing brains of young downstream (about 17 cm in length) and mature upstream (about 85 cm in length) migrating adult sea lampreys (Figure 3). The general distribution of *PmGalP+* cells in young and mature adult lampreys is similar and therefore the description below of *PmGalP+* cells in adult lampreys is based on our analysis of both young and mature animals.

As in larvae, the most rostral *PmGalP+* population was observed in the periventricular area of the septum (Figures 3B,F). The preoptic nucleus of adult sea lampreys also contained strongly stained *PmGalP+* cells (Figures 3C,G). Interestingly, a new and conspicuous population of *PmGalP+* cells was found dispersed in the adult telencephalon in a region ranging from the area lateral to the dorsal part of the preoptic nucleus to the ventral part of the lateral pallium (Figures 3B,C,G,H). In adult lampreys, some weakly stained *PmGalP+* cells were also observed in the characteristic cell band of the striatum (Figures 3B,G,H).

In the adult sea lamprey diencephalon, a *PmGalP+* population was also found in the prethalamus. These *PmGalP+* cells were found in the rostral prethalamus as in larvae, but they also extended more caudally in adults (Figures 3D,E,J). Laterally displaced *PmGalP+* cells were also present in the prethalamus (Figures 3D,E,J). These displaced cells of the prethalamus appeared to be in continuity with those of the telencephalon (see previous paragraph). In the dorsal and ventral hypothalamus of adult lampreys, a large group of *PmGalP+* cells was observed in periventricular layers around both the post-optic and the infundibular recesses. In the ventral hypothalamus, these cells were strongly stained and occupied three to four compact rows of cells. In the dorsal hypothalamus, we observed the presence of fewer *PmGalP+ cells* and these were less densely packed (Figures 3E,J,K). As in larvae, laterally displaced *PmGalP+* cells were also observed in the hypothalamus, although these cells were more numerous than in larvae (Figures 3E,I–K).

## Discussion

Galanin is a 29-residue neuropeptide in vertebrates (30 residues in humans) with numerous endocrine activities. Exogenously administered galanin has many biological actions, including inhibition of acetylcholine and insulin release, stimulation of feeding, modulation of spinal nociceptive flexor reflexes, inhibition of gastric acid secretion and reduction of alcohol consumption (Ch’ng et al., 1985; Amiranoff et al., 1989; Xu et al., 1990, 1995; Crawley, 1995; Kask et al., 1995; Millón et al., 2019). The amino acid sequence of gnathostome galanins is in general very conserved, as they only differ in five amino acid residues. Notably, most of these differences are in the C-terminal region from residues 16 to 30, whereas residues 1–15 are highly conserved (Fisone et al., 1989; Land et al., 1991; Mensah et al., 2010). In this study, we report the identification of a galanin precursor in the agnathan *P. marinus* (PmGalP). PmGalP contains a predicted C-terminally amidated peptide comprising 26 residues, which is 3 to 4 residues shorter than galanins found in other vertebrates. An alignment of the *P. marinus* galanin with galanins from gnathostomes shows that the lamprey galanin is the most divergent of the sequences reported thus far in vertebrates, with several non-conservative amino acid substitutions. Furthermore, *P. marinus* galanin does not align completely with gnathostome galanins in the C-terminal region, due its shorter length. However, the first thirteen residues are identical to those in gnathostome galanins and residues 15 to 21 comprise a combination of conserved and non-conserved residues (Figure 1B).

Interestingly, receptor binding assays and *in vivo* pharmacological experiments have demonstrated that the N-terminal region of galanins is the most important region for the activation of galanin receptors and subsequent biological actions. Experiments using different fragments of galanins demonstrated that synthetic galanin containing only the first 15 or 16 residues, GAL(1–15) and GAL(1–16), binds to galanin receptors with affinity in the nanomolar range, with a fivefold lower affinity compared to full-length galanin. In contrast, synthetic galanin containing residues 17–29 of galanin, GAL(17–29), has 10,000-fold lower affinity compared to galanin. This suggests that the C-terminal residues 17–29 contribute very little to receptor binding and activation (Fisone et al., 1989; Lagny-Pourmir et al., 1989; Gallwitz et al., 1990). Furthermore, *in vivo* analysis of the inhibitory effects of galanin on gastric acid secretion in rats revealed that N-terminal fragments of galanin (GAL 1–10) and (GAL 1–15) retain approximately 60% of the activity of full-length galanin, whilst a C-terminal fragment (GAL 15–29) had no bioactivity when tested at the same dose ranges as galanin and the fragment (GAL 9–29) retained only 5% of activity of full-length galanin (Rossowski and Coy, 1989; Mungan et al., 1992). These findings are consistent with the finding that the N-terminal 13-amino acid residues of galanin are conserved in vertebrates, including *P. marinus*, whereas the C-terminal region of galanins is much more variable and most notably in *P. marinus*. Therefore, the divergence in the C-terminal region of *P. marinus* galanin by comparison with gnathostome galanins likely reflects lack of selection pressure because this region is less important than the N-terminal region for receptor activation and bioactivity.

Previous studies on the organization of the galaninergic system in the CNS of lampreys were performed only in adults and using antibodies generated against porcine galanin (Buchanan et al., 1987; Jiménez et al., 1996; Yáñez et al., 1999). Here, we generated specific riboprobes against the *PmGalP* and analyzed its expression in the CNS of larval and adult sea lampreys using *in situ* hybridisation. This confirmed the presence of previously reported (Jiménez et al., 1996) galanin-like-ir periventricular cell populations of the sea lamprey prosencephalon (septal, hypothalamic and prethalamic populations) and galanin-like-ir laterally migrated telencephalic cells. However, Jiménez et al. (1996) used an outdated neuroanatomical nomenclature in their immunohistochemical study, with the septal region identified as the *nucleus commissurae anterior* by these authors. Our analysis using *in situ* hybridisation also identified strong *PmGalP* expression in the preoptic area in continuation with the septal population, the presence of weakly stained striatal *PmGalP+* cells and the presence of laterally migrated *PmGalP+* cells in the prethalamus and hypothalamus. These galaninergic populations were not previously reported by Jiménez et al. (1996) in their immunohistochemical study. The reasons for these discrepancies might be related to the sensitivity of the porcine antibodies used or to the differential accumulation of *PmGalP* transcripts and mature galanin peptide in the soma and fibers of galaninergic neurons. In addition, we extended our analyses to the larval brain showing that most of the galaninergic populations are already present before the metamorphosis, with the exception of the laterally migrated and striatal cells of the telencephalon, which were only present in adult lampreys.

The advantage of previous immunohistochemical studies is that they revealed the presence of extensive galanin-like-ir innervation of the brain (Jiménez et al., 1996), parapineal organ (Yáñez et al., 1999), and spinal cord (Buchanan et al., 1987). Our study confirms the lack of galaninergic cells in the spinal cord and brainstem, which suggests that the galanin-like-ir fibers of the lamprey spinal cord reported by Buchanan et al. (1987) must be of hypothalamic origin. The hypothalamus is the only brain region with *PmGalP* expressing neurons that also contains descending neurons that project to the spinal cord in lampreys (Barreiro-Iglesias et al., 2008). This should be experimentally confirmed in future hodological studies.

As noted by Jiménez et al. (1996), comparison with other vertebrates shows that the distribution of galaninergic neuronal populations in lampreys is similar to that of jawed fishes, since in both groups galanin-expressing neurons are mainly restricted to the prosencephalon. This is in striking contrast to amphibians, reptiles, birds and mammals, in which galaninergic cell populations are present also in the mesencephalon and rhombencephalon (see section “Introduction”). For example, in the brainstem of mammals, including humans, galanin expression is prominent in the locus coeruleus (Melander et al., 1986; Holets et al., 1988; Xu et al., 1998; Le Maître et al., 2013). Interestingly, tyrosine hydroxylase *in situ* hybridization and immunohistochemical studies indicate that lampreys do not have a locus coeruleus (Pierre et al., 1997; Barreiro-Iglesias et al., 2010), which suggests that these features evolved after the split of jawless and jawed vertebrates. So, evolution of the galaninergic system in vertebrates involved an increase in the number of mesencephalic and brainstem populations. Other neuronal systems, as serotonergic (Parent, 1984; Pierre et al., 1992) and glycinergic (Villar-Cerviño et al., 2008) systems, have also evolved with an increase in caudal populations. In contrast, present and previous results show that the presence of septal and hypothalamic galaninergic neuronal populations is a highly conserved character in all vertebrates (Goodson et al., 2004; Adrio et al., 2005). The galaninergic septal neurons have been implicated in the regulation of social behavior in birds and mammals (Goodson et al., 2004), whereas galaninergic hypothalamic neurons are mainly implicated in the regulation of feeding in fishes and mammals (Leibowitz et al., 1998; Sahu, 1998; Volkoff et al., 2005). Interestingly, both larval and adult lampreys have *PmGalP+* neurons in their septum and hypothalamus. Therefore, the lamprey would be an interesting vertebrate model to investigate the roles of galanin in these brain regions in context of very different developmental stages in terms of social and feeding behaviors. Our study provides a molecular and neuroanatomical basis for future functional studies on the role of galanin and GAMP in these and other brain regions of lampreys.

## Data Availability

The datasets generated for this study can be found in GenBank under accession number MK977616.

## Ethics Statement

This animal study was reviewed and approved by the Bioethics Committee at the University of Santiago de Compostela and the Consellería do Medio Rural e do Mar of the *Xunta de Galicia* (License Ref. JLPV/IId).

## Author Contributions

DS-C, LY-G, FL, CC-F, and HK contributed to the acquisition of experimental data. DS-C, LY-G, ME, RA, MR, and AB-I contributed to the data analysis and interpretation, and drafting of the manuscript. AB-I contributed to the concept and design of the study. All authors have approved the final manuscript.

## Conflict of Interest Statement

The authors declare that the research was conducted in the absence of any commercial or financial relationships that could be construed as a potential conflict of interest.

## Footnotes

1 www.ch.embnet.org/software/BOX_form.html

## Supplementary Material

FIGURE S1 Alignment of selected galanin precursors from vertebrates used for the identification of signal peptides (underlined in blue), mature peptides (underlined in red), and galanin-associated peptides (underlined in purple). Conserved residues are highlighted. Conservation in more than 70% of sequences is highlighted in black, conservative substitutions are highlighted in gray. Species names are as follows: Hsap (*Homo sapiens*), Btau (*Bos taurus*), Rnor (*Rattus norvegicus*), Sscr (*Sus scrofa*), Ggal (*Gallus gallus*), Asin (*Alligator sinensis*), Lcha (*Latimeria chalumnae*), Srhi (*Sinocyclocheilus rhinocerous*), Drer (*Danio rerio*), Amex *(Astyanax mexicanus)*, Locu *(Lepisosteus oculatus)*, Rtyp *(Rhincodon typus)*, Cmil (*Callorhinchus milii*), and Pmar (*Petromyzon marinus*). Accession numbers are shown next to the names.

FILE S1 Sequences used for the phylogenetic reconstruction in Figure 1C.

## Abbreviations

B3: rhombencephalic Müller cell 3
Ch: optic chiasm
DCN: dorsal column nucleus
DHyp: dorsal hypothalamus
dLP, lateral pallium: dorsal part
fr: fasciculus retroflexus
Ha: habenula
Hyp: hypothalamus
IS: isthmus
lHa: left habenula
LHyp: lateral hypothalamus
LP: lateral pallium
M1-3: giant Müller cells 1 to 3
Ma: Mauthner neuron
MI, giant isthmic neuron (i.e.: I1 neuron)
MLFn: nucleus of the medial longitudinal fascicle
MP: medial pallium
NH: neurohypophysis
OB: olfactory bulb
ON: optic nerve
OT: optic tectum
ot: optic tract
P: pineal organ
PC: posterior commissure
PO: preoptic nucleus
PoC: postoptic commissure nucleus
PoR: postoptic recess
PT: pretectum
PTh: prethalamus
PTN: posterior tubercle nucleus
Rh: rhombencephalon
rHa: right habenula
SC: spinal cord
ShL: subhippocampal lobe
Sp: septum
Str: striatum
Th: thalamus
TS: torus semicircularis
VHyp: ventral hypothalamus
vLP, lateral pallium: ventral part
Vm: trigeminal motor nucleus
Xm: vagal motor nucleus
zl: zona limitans intrathalamica.
