# Supplementary figures and images for: Galanin in an Agnathan: Precursor Identification and Localisation of Expression in the Brain of the Sea Lamprey Petromyzon marinus
Source: Front Neuroanat. 2019 Sep 13;13:83. doi: 10.3389/fnana.2019.00083 (PMC6753867; doi:10.3389/fnana.2019.00083)

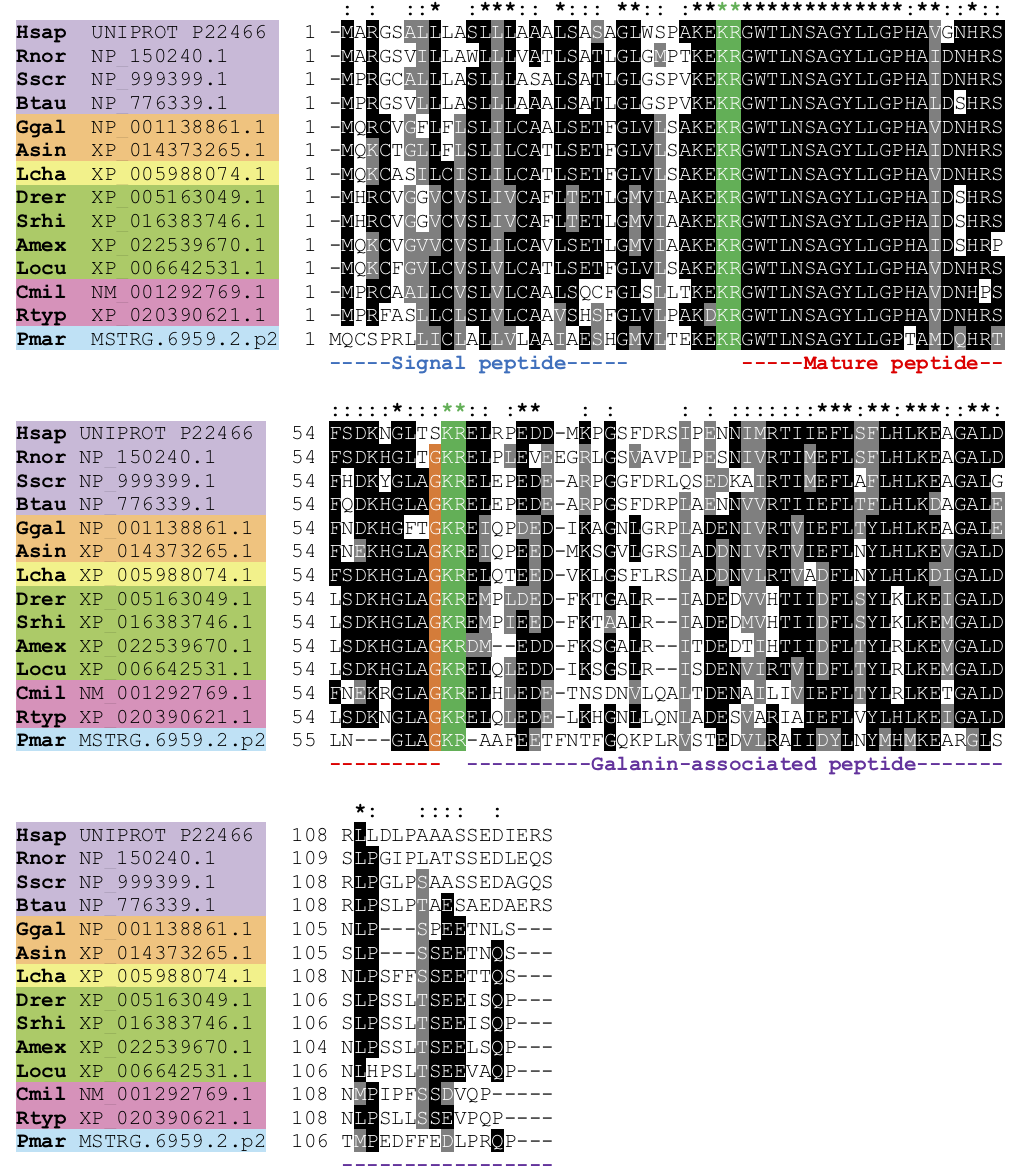

Supplement: FIGURE S1 — Alignment of selected galanin precursors from vertebrates used for the identification of signal peptides (underlined in blue), mature peptides (underlined in red), and galanin-associated peptides (underlined in purple). Conserved residues are highlighted. Conservation in more than 70% of sequences is highlighted in black, conservative substitutions are highlighted in gray. Species names are as follows: Hsap (Homo sapiens), Btau (Bos taurus), Rnor (Rattus norvegicus), Sscr (Sus scrofa), Ggal (Gallus gallus), Asin (Alligator sinensis), Lcha (Latimeria chalumnae), Srhi (Sinocyclocheilus rhinocerous), Drer (Danio rerio), Amex (Astyanax mexicanus), Locu (Lepisosteus oculatus), Rtyp (Rhincodon typus), Cmil (Callorhinchus milii), and Pmar (Petromyzon marinus). Accession numbers are shown next to the names. [file Image_1.TIF]
