# Supplementary material for: Galanin in an Agnathan: Precursor Identification and Localisation of Expression in the Brain of the Sea Lamprey Petromyzon marinus
Source: Front Neuroanat. 2019 Sep 13;13:83. doi: 10.3389/fnana.2019.00083 (PMC6753867; doi:10.3389/fnana.2019.00083)
Supplement: FILE S1 — Sequences used for the phylogenetic reconstruction in Figure 1C. [file Data_Sheet_1.PDF]

Supplementary file
>sp|P22466|GALA_HUMAN Galanin peptides OS=Homo sapiens OX=9606 GN=GAL PE=1 SV=3
MARGSALLLASLLLAAALSASAGLWSPAKEKRGWTLNSAGYLLGPHAVGNHRSFSDKNGLTSKRELRP
EDDMKPGSFDRSIPENNIMRTIIEFLSFLHLKEAGALDRLLDLPAAASSEDIERS
>NP_150240.1 galanin peptides preproprotein [Rattus norvegicus]
MARGSVILLAWLLLVATLSATLGLGMPTKEKRGWTLNSAGYLLGPHAIDNHRSFSDKHGLTGKRELPL
EVEEGRLGSVAVPLPESNIVRTIMEFLSFLHLKEAGALDSLPGIPLATSSEDLEQS
>NP_776339.1 galanin peptides preproprotein [Bos taurus]
MPRGSVLLLASLLLAAALSATLGLGSPVKEKRGWTLNSAGYLLGPHALDSHRSFQDKHGLAGKRELEP
EDEARPGSFDRPLAENNVVRTIIEFLTFLHLKDAGALERLPSLPTAESAEDAERS
>NP_999399.1 galanin peptides preproprotein [Sus scrofa]
MPRGCALLLASLLLASALSATLGLGSPVKEKRGWTLNSAGYLLGPHAIDNHRSFHDKYGLAGKRELEP
EDEARPGGFDRLQSEDKAIRTIMEFLAFLHLKEAGALGRLPGLPSAASSEDAGQS
>XP_014373265.1 galanin peptides isoform X2 [Alligator sinensis]
MQKCTGLLFLSLILCATLSETFGLVLSAKEKRGWTLNSAGYLLGPHAIDNHRSFNEKHGLAGKREIQP
EEDMKSGVLGRSLADDNIVRTVIEFLNYLHLKEVGALDSLPSSEETNQS
>XP_005988074.1 PREDICTED: galanin peptides isoform X1 [Latimeria chalumnae]
MQKCASILCISLILCATLSETFGLVLSAKEKRGWTLNSAGYLLGPHAVDNHRSFSDKHGLAGKRELQT
EEDVKLGSFLRSLADDNVLRTVADFLNYLHLKDIGALDNLPSFFSSEETTQS
>NP_001138861.1 galanin isoform 2 precursor [Gallus gallus]
MQRCVGFLFLSLILCAALSETFGLVLSAKEKRGWTLNSAGYLLGPHAVDNHRSFNDKHGFTGKREIQP
DEDIKAGNLGRPLADENIVRTVIEFLTYLHLKEAGALENLPSPEETNLS
>XP_006642531.1 PREDICTED: galanin peptides isoform X2 [Lepisosteus oculatus]
MQKCFGVLCVSLVLCATLSETFGLVLSAKEKRGWTLNSAGYLLGPHAVDNHRSLSDKHGLAGKRELQL
EDDIKSGSLRISDENVIRTVIDFLTYLRLKEMGALDNLHPSLTSEEVAQP
>XP_005163049.1 galanin peptides isoform X1 [Danio rerio]
MHRCVGGVCVSLIVCAFLTETLGMVIAAKEKRGWTLNSAGYLLGPHAIDSHRSLSDKHGLAGKREMPL
DEDFKTGALRIADEDVVHTIIDFLSYLKLKEIGALDSLPSSLTSEEISQP
>XP_022539670.1 galanin peptides isoform X2 [Astyanax mexicanus]
MQKCVGVVCVSLILCAVLSETLGMVIAAKEKRGWTLNSAGYLLGPHAIDSHRPLSDKHGLAGKRDMED
DFKSGALRITDEDTIHTIIDFLTYLRLKEVGALDNLPSSLTSEELSQP
>XP_016383745.1 PREDICTED: galanin peptides isoform X1 [Sinocyclocheilus rhinocerous]
MHRCVGGVCVSLIVCAFLTETLGMVIAAKEKRGWTLNSAGYLLGPHAIDSHRSLSDKHGLAGKREMPI
EEDFKTAALRISDEDVVYTIIDFLSYLKLKEMGALDSLPSSLTSEEISQP
>XP_020390621.1 galanin peptides [Rhincodon typus]
MPRFASLLCLSLVLCAAVSHSFGLVLPAKDKRGWTLNSAGYLLGPHAVDNHRSLSDKNGLAGKRELQL
EDELKHGNLLQNLADESVARIAIEFLVYLHLKEIGALDNLPSLLSSEVPQP
>NM_001292769.1 Callorhinchus milii galanin and GMAP prepropeptide (gal), mRNA
MPRCAALLCVSLVLCAALSQCFGLSLLTKEKRGWTLNSAGYLLGPHAVDNHPSFNEKRGLAGKRELHL
EDETNSDNVLQALTDENAILIVIEFLTYLRLKETGALDNMPIPFSSDVQP
>MSTRG.6959.2.p2 LAMPREY Petromyzon marinus
MQCSPRLLICLALLVLAAIAESHGMVLTEKEKRGWTLNSAGYLLGPTAMDQHRTLNGLAGKRAAFEET
FNTFGQKPLRVSTEDVLRAIIDYLNYMHMKEARGLSTMPEDFFEDLPRQP
>NM_001278965.1 Ciona intestinalis galanin-like peptide (ci-galp), mRNA (OUTGROUP, only used for the phylogenetic analysis).
MNSFGRYTFSLAVVLYISIVLCAENSEAATAKRPFRGQG**GWTLNSVGY**NAGLGALRKLFEKRDGSSLD
VESMPLDNEEMENLAKDFALFLEVKESGLLGPRMLRCILSRNDQVMDSMSEM
